# Comparison of the continuation and discontinuation of perioperative antiplatelet therapy in laparoscopic surgery for colorectal cancer: A retrospective, multicenter, observational study (YCOG 1603)

**DOI:** 10.1002/ags3.12387

**Published:** 2020-10-28

**Authors:** Hiroki Ohya, Jun Watanabe, Yusuke Suwa, Kazuya Nakagawa, Hirokazu Suwa, Mayumi Ozawa, Atsushi Ishibe, Chikara Kunisaki, Itaru Endo

**Affiliations:** ^1^ Department of Surgery Gastroenterological Center Yokohama City University Medical Center Yokohama Japan; ^2^ Department of Gastroenterological Surgery Yokohama City University Graduate School of Medicine Yokohama Japan; ^3^ Department of Surgery Yokosuka Kyosai Hospital Yokosuka Japan

**Keywords:** antiplatelet therapy, cardiovascular events, colorectal cancer, hemorrhagic complication, laparoscopic surgery

## Abstract

**Aim:**

The present study aimed to examine the effect of continuing antiplatelet therapy in the perioperative period for patients undergoing laparoscopic resection for colorectal cancer who had received preoperative antiplatelet therapy.

**Methods:**

This retrospective, multicenter, observational study included patients who underwent laparoscopic surgery for colorectal cancer between January 2011 and May 2020. The study population was limited to patients who used antiplatelet therapy preoperatively.

**Results:**

A total of 214 colorectal cancer patients who received antiplatelet therapy preoperatively were included in the present study. Eighty‐nine patients underwent surgery under the continuation of antiplatelet therapy, and 125 patients underwent surgery under the discontinuation of antiplatelet therapy before surgery. There were no significant differences between the two groups with regard to intraoperative blood loss (*P* = .889), intraoperative blood transfusion (*P* = 1.000), and conversion to laparotomy (*P* = 1.000). There were no significant differences between the two groups in the incidence of postoperative hemorrhagic complications (Clavien‐Dindo Grade ≥II, *P* = .453; Grade ≥III, *P* = .572) or three‐point major adverse cardiovascular events (*P* = .268). However, there were two cases of postoperative non‐fatal stroke in the discontinued antiplatelet therapy group.

**Conclusions:**

The present study revealed that there were no significant differences in the surgical outcomes and postoperative complications between colorectal cancer patients who underwent laparoscopic resection with the continuation of antiplatelet therapy in the perioperative period and those in whom antiplatelet therapy was discontinued during the perioperative period. From the viewpoint of cardiovascular and cerebrovascular risk, it may be better for patients undergoing laparoscopic surgery for colorectal cancer to continue antiplatelet therapy. This study was registered with the Japanese Clinical Trials Registry as UMIN000038707 (http://www.umin.ac.jp/ctr/index.htm).

## Graphical abstract

The present study aimed to examine the effect of continuing antiplatelet therapy in the perioperative period for patients undergoing laparoscopic resection for colorectal cancer who had received preoperative antiplatelet therapy. There were no significant differences between the two groups with regard to intraoperative blood loss (*P* = .889), postoperative hemorrhagic complications. However, there were two cases of postoperative non‐fatal stroke in the discontinuation of antiplatelet therapy group. It may be better for patients undergoing laparoscopic surgery for colorectal cancer to continue antiplatelet therapy.

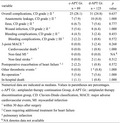

## 1 INTRODUCTION

Antiplatelet therapy (APT) has been widely used for the secondary prevention of cardiovascular diseases, such as coronary artery disease and cerebrovascular disease, including cerebral infarction and transient ischemic attack.^1^, ^2^ In recent years, the effects of aspirin on the secondary prevention of these diseases have become clear, and the proportion of patients who receive APT at the time of surgery has been increasing.^3^, ^4^ Annually, 10% of patients receiving antithrombotic therapy (ATT), including APT and anticoagulation therapy (ACT), undergo surgery or other invasive procedures that require the temporary discontinuation of therapy.^3^ However, excessive perioperative ATT withdrawal may put patients at risk for thromboembolism.^5^ Regarding the continuation or discontinuation of APT in the perioperative period of non‐cardiac surgery, the risks and benefits have been considered to differ depending on the background of the patient undergoing APT and the surgical procedure being performed.^6^, ^7^ However, there is limited information about the cardiovascular and/or cerebrovascular disease risk of each patient population and the bleeding risk associated with each surgical procedure. Thus, there has been no consensus on whether APT should be continued or discontinued during the perioperative period.^8^, ^9^

Laparoscopic surgery is now widely performed for colorectal cancer (CRC)^10^; however, there is no consensus regarding the continuation or discontinuation of APT during these operations. Laparoscopic colorectal resection is generally considered to be associated with an intermediate risk of bleeding.^11^ In recent years, there have been several reports about laparoscopic colorectal resection under continuous APT; however, the debate is still insufficient.^12^ Postoperative hemorrhagic complications are potentially fatal complications; similarly, postoperative cardiovascular and/or cerebrovascular events (CVE) represent serious and potentially fatal complications.

The present study aimed to examine the effect of continuing APT in the perioperative period for patients undergoing laparoscopic resection for CRC who had received preoperative APT.

## 2 METHODS

### 2.1 Patient selection, antiplatelet therapy, and outcomes

This retrospective, multicenter, observational study was conducted within the framework of the Yokohama Clinical Oncology Group (YCOG) in Japan. Patients who underwent laparoscopic surgery for CRC were recruited from two institutions of the YCOG between January 2011 and May 2020. This study protocol was approved by the Ethical Advisory Committee of Yokohama City University Medical Center and the institutional review board of each participating hospital and conformed to the provisions of the Declaration of Helsinki. Patient data were collected from clinical reports from each institution. The study was registered with the Japanese Clinical Trials Registry as UMIN000038707 (http://www.umin.ac.jp/ctr/index.htm).

In this study, we targeted patients who were using aspirin, cilostazol, clopidogrel, ticlopidine, or prasugrel as APT before surgery. Patients who received antiplatelet drugs other than the five abovementioned drugs were excluded from this study. The eligibility criteria of this study were as follows: (a) CRC patients >20 years of age (no upper age limit was applied), (b) who underwent laparoscopic surgery, (c) and who had received at least one of the five abovementioned APT drugs before surgery. The exclusion criteria of this study were as follows: (a) patients who underwent open or robotic surgery, (b) patients with multiple primary cancers, and (c) patients who underwent combined resection of other organs.

The decision whether to continue APT (c‐APT group) or discontinue APT (d‐APT group) during the perioperative period was made by the attending surgeon. In the c‐APT group, the procedure for continuation of APT was as follows: (a) in patients receiving preoperative aspirin monotherapy, aspirin was continued; (b) in patients receiving preoperative drugs other than aspirin, the drug was substituted with aspirin; (c) patients who received preoperative dual antiplatelet therapy (DAPT) including aspirin continued APT with aspirin monotherapy; (d) patients who received preoperative DAPT without aspirin, it was selected to replace with aspirin and continue with aspirin only or continue with DAPT. In the c‐APT group, aspirin was taken orally until the morning of surgery and was resumed from the day after surgery. For patients in the d‐APT group, either (a) the discontinuation of APT or (b) preoperative heparin bridging were selected based on the judgment of the attending surgeon. The durations of the preoperative discontinuation of antiplatelet drugs were 3 days for cilostazol, 7 days for aspirin and ticlopidine, 14 days for clopidogrel and prasugrel. In the cases of bridge therapy, the bridging agent (aspirin or heparin) was started on the day that each APT agent was discontinued. In the d‐APT group, postoperative APT was resumed at the discretion of the attending surgeon.

Antithrombotic therapy for preventing pulmonary embolism (PE) were not routinely performed after surgery in our institutions. ATT was only performed when thrombotic complications including deep venous thrombosis (DVT) and PE were observed before and after surgery.

The primary outcome of this study was the incidence of intraoperative and postoperative hemorrhagic complications. The secondary outcomes of this study were the operation time, intraoperative blood loss, intraoperative blood transfusion rate, the rate of conversion to laparotomy, the length of postoperative hospital stay, all postoperative complications within 30 days (Clavien‐Dindo classification [CD] grade ≥II), major adverse cardiovascular events (MACE), the exacerbation of postoperative heart failure, the reoperation rate, and in‐hospital death. In addition, the following preoperative clinical data were collected: age, body mass index (BMI), American Society of Anesthesiologists class (ASA‐class), Eastern Cooperative Oncology Group performance status (ECOG‐PS), smoking habits, comorbidities (including diabetes, hypertension, and chronic heart failure), preoperative hemoglobin (Hb) level, preoperative platelets (PLT) count, preoperative prothrombin time (PT), preoperative activated partial thromboplastin time (APTT), preoperative brain natriuretic peptide (BNP), preoperative blood sugar (BS) level, prognostic nutritional index (PNI), tumor location, tumor diameter, and UICC TNM stage. In this study, classical three‐point MACE was used for the assessment of MACE. Classical three‐point MACE was defined as a composite of non‐fatal stroke, non‐fatal myocardial infarction (MI), and cardiovascular death.

### 2.2 Surgical procedure

Laparoscopic surgery for CRC was usually performed with five ports. Lymph node dissection and colon or rectum mobilization, including central vascular ligation of the artery and vein, were all performed laparoscopically. Sigmoidectomy and rectal resection with anastomosis using the double stapling technique (DST) were performed with two 12‐mm ports and three 5‐mm ports, and statistical analyses colectomy with antiperistaltic side‐to‐side anastomosis (functional end‐to‐end anastomosis: FEEA) was performed with one 12‐mm port and four 5‐mm ports. DST reconstruction was performed intracorporeally using an ILS^™^ (Ethicon Endo‐Surgery) circular stapler. FEEA reconstruction was performed extracorporeally using an Echelon Flex™ (Ethicon Endo‐Surgery), a Signia, or an i‐Drive (Medtronic) stapler.

### 2.3 Statistical analysis

Categorical variables were expressed as the frequency and proportion (%), and numerical data were presented as the median and interquartile range (IQR). All statistical analyses were performed using EZR (Saitama Medical Center, Jichi Medical University, Saitama, Japan^13^), which is a graphical user interface for R (The R Foundation for Statistical Computing). The Mann‐Whitney *U* test, the Fisher's exact test, and Pearson's chi‐squared test were performed for comparisons between independent groups when appropriate. *P* values of <.05 were considered to indicate statistical significance.

## 3 RESULTS

A total of 214 patients with CRC who had received APT preoperatively between January 2011 and May 2020 were included in this study. Eighty‐nine patients underwent surgery under the continuation of APT (c‐APT group), and 125 patients underwent surgery under the discontinuation of APT before surgery (d‐APT group). The patient and oncological characteristics are summarized in Table 1. The percentage of male patients in the c‐APT group was significantly greater than that in the d‐APT group (72/89 [80.9%] in the c‐APT group vs 85/125 [68.0%] in the d‐APT group; *P* = .042). The preoperative ECOG‐PS in the c‐APT group was significantly worse than that in the d‐APT group (*P* = .004). There were not significant differences in age, BMI, ASA‐class, smoking habits, presence of diabetes, presence of hypertension, presence of chronic heart failure, Hb level, PLT count, PT, APTT, BNP, BS level, PNI, tumor location, tumor diameter, or UICC TNM stage between the two groups.

**Table 1:** Patients' and oncological characteristics

Variable	c‐APT Gr	d‐APT Gr	*P* value
	n = 89	n = 125	
Age (y)	75 [70‐79]	76 [70‐81]	.553
Male sex	72 (80.9)	85 (68.0)	.042
BMI (kg/m^2^)	23.6 [21.5‐25.1]	23.6 [21.0‐25.9]	.752
ASA‐class			
I	2 (2.2)	3 (2.4)	.959
II	63 (70.8)	88 (70.4)	
III	24 (27.0)	33 (26.4)	
IV	0 (0.0)	1 (0.8)	
ECOG‐PS			
0	50 (56.2)	95 (76.0)	.004
1	35 (39.3)	24 (19.2)	
2	2 (2.2)	6 (4.8)	
3	2 (2.2)	0 (0.0)	
Smoking habits	46 (51.7)	73 (58.4)	.402
Diabetes	33 (37.1)	34 (27.2)	.137
Hypertension	62 (69.7)	76 (60.8)	.195
Chronic heart failure	5 (5.6)	5 (4.0)	.745
Hb (g/dL)^a^	11.9 [10.6‐13.1]	12.2 [10.6‐13.3]	.899
PLT (×10^4^ cells/mm^3^)^a^	22.6 [19.8‐26.0]	22.0 [17.3‐26.6]	.242
PT‐INR^a^	1.00 [0.97‐1.10]	1.00 [0.90‐1.10]	.316
APTT (s)^a^	29.9 [28.0‐32.2]	29.2 [27.4‐31.8]	.489
BNP (pg/dL)^a^	51.5 [25.4‐134.7]	49.8 [27.8‐107.0]	.916
BS (mg/dL)^a^	110 [100‐133]	108 [97‐120]	.164
HbA1c (%)^a^	6.1 [5.7‐6.5]	5.9 [5.5‐6.4]	.068
PNI	47.8 [43.6‐52.0]	48.5 [45.0‐51.6]	.284
Tumor location			
Colon	62 (69.7)	85 (68.0)	.881
Rectum	27 (30.3)	40 (32.0)	
Tumor diameter (mm)	35 [24‐50]	35 [25‐50]	.705
UICC TNM stage			
0‐I	30 (33.7)	40 (32.0)	.520
II	29 (32.6)	40 (32.0)	
III	29 (32.6)	38 (30.4)	
IV	1 (1.1)	7 (5.6)	
Emergency case	1 (1.1)	2 (1.6)	1.000

Note

Numerical data are indicated as medians. Values in parentheses are percentages and values in brackets are IQR; first quartile‐third quartile.

Abbreviations: (10 × Albumin [g/dL]) + (0.005 × Total lymphocyte count); APTT, activated partial thromboplastin time; ASA, American Society of Anesthesiologists; BMI, body mass index, calculated as weight in kilograms divided by height in meters squared; BNP, brain natriuretic peptide; BS, blood sugar level; c‐APT Gr, antiplatelet therapy continuation group; d‐APT Gr, antiplatelet therapy discontinuation group; ECOG, Eastern Cooperative Oncology Group; Hb, hemoglobin; HbA1c, hemoglobin A1c; PLT, platelet; PNI, prognostic nutritional index, calculated as follows; PS, performance status; PT, prothrombin time.

^a^ Values are preoperative data.

The details of APT are summarized in Table 2. Regarding the indication of APT, there were significantly more cases of coronary artery disease in the c‐APT group than in the d‐APT group (c‐APT 67/89 [75.3%] vs d‐APT 66/125 [52.8%]; *P* = .001) and there were significantly more cases in which APT was administered for primary prevention in the d‐APT group than in the c‐APT group (c‐APT 0/89 [0%] vs d‐APT 21/125 [16.8%]; *P* < .001). Regarding the details of APT, aspirin was the most frequently administered agent in both groups. In many patients who received DAPT, clopidogrel was used in addition to aspirin. There were many cases where cilostazol was used for cerebral infarction (these data were not shown). In all cases in which prasugrel was administered, it was as part of DAPT, and all cases underwent surgery under continuous aspirin monotherapy. There was no significant difference between the two groups in the proportion of cases in which APT was combined with anticoagulation therapy (ACT; c‐APT 6/89 [6.7%] vs d‐APT 17/125 [13.6%]; *P* = .123). In the c‐APT group, 16 patients (18.0%) received aspirin as a substitute for another agent preoperatively and continued using aspirin in the perioperative period. In the d‐APT group, 46 patients (36.8%) received heparin bridging therapy. In the c‐APT group, 87 patients (97.8%) continued aspirin monotherapy and two patients (2.2%) continued DAPT.

**Table 2:** Details about antiplatelet therapy

Variable	c‐APT Gr	d‐APT Gr	*P* value
	n = 89	n = 125	
Indication of APT			
Coronary artery disease	67 (75.3)	66 (52.8)	.001
Peripheral arterial disease	1 (1.1)	5 (4.0)	.404
Valvular disease	3 (3.4)	6 (4.8)	.738
Aortic aneurysm/ Aortic dissection	3 (3.4)	1 (0.8)	.310
Cerebral infarction	22 (24.7)	24 (19.2)	.399
Transient ischemic attack	3 (3.4)	5 (4.0)	1.000
Primary prevention	0 (0.0)	21 (16.8)	<.001
Details of APT			
Aspirin	71 (79.8)	98 (78.4)	.866
Clopidogrel	19 (21.3)	28 (22.4)	1.000
Cilostazol	9 (10.1)	13 (10.4)	1.000
Ticlopidine	1 (1.1)	4 (3.2)	.405
Prasugrel	6 (6.7)	0 (0.0)	.005
DAPT	23 (25.8)	18 (14.4)	.052
Combined use of ACT	6 (6.7)	17 (13.6)	.123
Aspirin bridging	16 (18.0)	1 (0.8)	<.001
Heparin bridging	2 (2.2)	46 (36.8)	<.001
Continuation of aspirin monotherapy	87 (97.8)	0 (0.0)	<.001
Continuation of DAPT	2 (2.2)	0 (0.0)	.172

Note

Numerical data are indicated as medians. Values in parentheses are percentages and values in brackets are IQR: first quartile‐third quartile.

Abbreviations: ACT, anticoagulation therapy; APT, antiplatelet therapy; c‐APT Gr, antiplatelet therapy continuation Group; d‐APT Gr, antiplatelet therapy discontinuation group; DAPT, dual antiplatelet therapy;

The surgical outcomes are summarized in Table 3. There were no significant differences between the two groups in intraoperative blood loss (c‐APT 10 mL [5‐50] vs d‐APT 10 mL [5‐50]; *P* = .889), the rate of intraoperative blood transfusion (3/89 [3.4%] vs 4/125 [3.2%]; *P* = 1.000), or the rate of conversion to laparotomy (1/89 [1.1%] vs 2/125 [1.6%]; *P* = 1.000]). In addition, there were no significant differences between the two groups in terms of the operation time (189 minutes [149‐256] vs 185 minutes [151‐230]; *P* = .872), postoperative day of first flatus (1.0 [1.0‐2.0] vs 1.0 [1.0‐2.0]; *P* = .674), postoperative day of first defecation (3.0 [1.0‐4.0] vs 2.0 [1.0‐3.0]; *P* = .243), postoperative meal start date (3.0 [2.0‐4.0] vs 3.0 [2.0‐4.0]; *P* = .864), or length of postoperative stay (8.0 [6.0‐14.0] vs 8.0 [7.0‐13.3]; *P* = .650).

**Table 3:** Surgical outcomes

Variables	Laparoscopic colectomy		*P* value	Laparoscopic rectal resection		*P* value
	c‐APT	d‐APT		c‐APT	d‐APT	
	n = 62	n = 85		n = 27	n = 40	
Operation time (min)	171 [139‐206]	173 [146‐206]	.835	263 [198‐296]	232 [186‐264]	.237
Intraoperative blood loss (mL)	5 [5‐22]	10 [5‐33]	.581	35 [9‐88]	18 [5‐52]	.223
Intraoperative blood transfusion	3 (4.8)	2 (2.4)	.650	0 (0.0)	2 (5.0)	.512
High ligation of central vessels for LND	48 (77.4)	60 (70.6)	.450	22 (81.5)	28 (70.0)	.394
Total wound length (mm)	45 [40‐55]	50 [40‐60]	.260	40 [35‐50]	45 [40‐50]	.060
Conversion to laparotomy	1 (1.6)	2 (2.4)	1.000	0 (0.0)	0 (0.0)	N*A*
Diverting stoma	1 (1.6)	2 (2.4)	1.000	11 (40.7)	17 (42.5)	1.000
Drain insertion	26 (41.9)	27 (31.8)	.227	24 (88.9)	39 (97.5)	.295
Postoperative day of drain removal (d)	4 [3‐6]	4 [4‐5]	.464	5 [4‐8]	5 [4‐7]	.798
Postoperative day of APT resumption (d)	1 [1‐1]	4 [2‐6]	<.001	1 [1‐1]	5 [3‐7]	<.001
Postoperative day of first flatus (d)	2 [1‐2]	2 [1‐2]	.623	1 [1‐1]	1 [1‐1]	.718
Postoperative day of first defecation (d)	3 [2‐4]	3 [2‐3]	.111	1 [1‐2]	1 [1‐2]	.707
Postoperative meals start date (d)	3 [2‐3]	2 [2‐4]	.937	3 [3‐5]	3 [3‐4]	.517
Length of postoperative stay (d)	8 [6‐10]	8 [6‐10]	.301	14 [8‐17]	12 [7‐20]	.819

Note

Numerical data are indicated as medians. Values in parentheses are percentages and values in brackets are IQR: first quartile‐third quartile.

Abbreviations: c‐APT Gr, antiplatelet therapy continuation Group; d‐APT Gr, antiplatelet therapy discontinuation group; LND, lymph node dissection; NA, not available.

The postoperative complications are presented in Table 4. The incidence of overall complications (CD ≥ II) within 30 days after surgery was 25/89 (28.1%) in the c‐APT group and 31/125 (24.8%) in the d‐APT group, and did not differ to a statistically significant extent (*P* = .637). Furthermore, the incidence of postoperative hemorrhagic complications in patients with CD ≥ II (4/89 [4.5%] vs 3/125 [2.4%]; *P* = .453) and CD ≥ III (2/89 [2.2%] vs 1/125 [0.8%]; *P* = .572) complications did not differ to a statistically significant extent. There was no significant difference in three‐point MACE between the two groups (*P* = .268); there were no cases of three‐point MACE in the c‐APT group, and only three cases (2.4%) in the d‐APT group. A postoperative exacerbation of heart failure was observed in two cases of the c‐APT group and one case of the d‐APT group (*P* = .572), and postoperative pulmonary thromboembolism was only observed in one case of the d‐APT group (*P* = 1.000). Reoperations within 30 days after surgery were performed in five cases in the c‐APT group and seven cases in the d‐APT group (*P* = 1.000). There was one in‐hospital death in each group. Regarding the details of in‐hospital death, the in‐hospital death in the c‐APT group was due to pan peritonitis after anastomotic leakage, while that in the d‐APT group occurred due to rupture of a thoracic aortic aneurysm.

**Table 4:** Postoperative complications

Variable	c‐APT Gr	d‐APT Gr	*P* value
	n = 89	n = 125	
Overall complications, CD grade ≥ II^a^	25 (28.1)	31 (24.8)	.637
Anastomotic leakage, CD grade ≥ II^a^	7 (7.9)	10 (8.0)	1.000
Ileus, CD grade ≥ II^a^	6 (6.7)	7 (5.6)	.777
Wound infection, CD grade ≥ II^a^	5 (5.6)	8 (6.4)	1.000
Bleeding complications, CD grade ≥ II^a^	4 (4.5)	3 (2.4)	.453
Bleeding complications, CD grade ≥ III^a^	2 (2.2)	1 (0.8)	.572
3‐point MACE^a^	0 (0.0)	3 (2.4)	.268
Cardiovascular death^a^	0 (0.0)	1 (0.8)	1.000
Non‐fatal MI^a^	0 (0.0)	0 (0.0)	NA
Non‐fatal stroke^a^	0 (0.0)	2 (1.6)	.512
Postoperative exacerbation of heart failure^a^, ^b^	2 (2.2)	1 (0.8)	.572
Other thrombotic events^a^	0 (0.0)	1^c^ (0.8)	1.000
Re‐operation^a^	5 (5.6)	7 (5.6)	1.000
In‐hospital death	1 (1.1)	1 (0.8)	1.000

Note

Numerical data are indicated as medians. Values in parentheses are percentages.

Abbreviations: c‐APT Gr, antiplatelet therapy continuation Group; CD, Clavien‐Dindo classification; d‐APT Gr, antiplatelet therapy discontinuation group; MACE, major adverse cardiovascular events; MI, myocardial infarction; NA, data not available.

^a^ Within 30 d after surgery.

^b^ Cases requiring additional treatment for heart failure.

^c^ Pulmonary infarction.

Regarding the cases of postoperative hemorrhagic complications of CD ≥ III, there were two cases in the c‐APT group and one case in the d‐APT. The postoperative clinical courses of these patients were as follows. The first case in the c‐APT group was a 62‐year‐old male who had been prescribed aspirin for coronary artery disease and continued perioperative aspirin. He was diagnosed with anastomotic leakage on postoperative day (POD) 3. Therefore, the detention of drain was continued. An emergency laparotomy was performed on POD 17 due to increased bloody drainage from drain and shock‐vital condition. Intraoperative findings showed intra‐abdominal hemorrhage from the bottom of the pelvis. After hemostasis, drain was removed on POD 33 and he was discharged on POD 70. The second case in c‐APT group was a 68‐year‐old male who had been prescribed DAPT for coronary artery disease and continued perioperative aspirin monotherapy. He had bloody stool on POD 7 after starting the meal on POD 5. He was diagnosed with anastomotic hemorrhage by endoscopy, and hemostasis was ablated. After that, he was discharged on POD 12 without any findings of rebleeding. The bleeding case in d‐APT was a 79‐year‐old male who had been prescribed aspirin for coronary artery disease and withdrawal of aspirin 7 days before surgery. He had bloody stool on POD 1 and needed a blood transfusion. He was diagnosed with anastomotic hemorrhage by endoscopy, and hemostasis was ablated. After that, he was discharged on POD 11 without any findings of rebleeding.

## 4 DISCUSSION

This retrospective multicenter study revealed that there was no significant difference in the surgical outcomes or postoperative complications between patients who underwent laparoscopic surgery for CRC with the continuation of APT in the perioperative period and those in whom APT was discontinued in the perioperative period. There was no significant difference in the incidence of intraoperative and postoperative hemorrhagic complications between the two groups. However, although the incidence did not differ to a statistically significant extent, there were two cases of postoperative non‐fatal stroke in patients in whom APT was discontinued during the perioperative period.

Several previous studies have shown that APT with agents such as aspirin is useful for the secondary prevention of cardiovascular and/or cerebrovascular disease.^1^, ^2^ Previously, aspirin was also prescribed for the primary prevention of CVE; however, in recent years there has been some skepticism regarding the use of aspirin for primary prevention.^14^ The benefits of APT over primary and secondary prevention of CVE had been always discussed in contradistinction to increased bleeding risk. Due to the conspicuous medicinal properties of the APT, adverse effects associated with bleeding tendency are always a problem with these medicines. Hemorrhagic complications, especially in surgical procedures, can sometimes be fatal. Thus, it has long been considered that APT should be discontinued before non‐cardiac surgery. However, the occurrence of CVE associated with the withdrawal of APT is not uncommon, and the occurrence of CVE after surgery may be deadly.

Laparoscopic surgery for CRC has often been reported to be associated with an intermediate risk of bleeding, like any other abdominal open surgery or laparoscopic surgery.^11^ Although some guidelines have been published, there is still no complete consensus on whether or not to continue APT.^15^ Some previous studies have reported that perioperative APT during surgery in various areas was not associated with an increase in hemorrhagic complications.^16^, ^17^, ^18^ Another study suggested that the continuation of APT reduced the incidence of MACE after surgery.^19^ However, these studies targeted the heterogeneous patient populations who were treated with several different surgical procedures, not a single procedure. Even though these procedures were in the same intermediate risk group, they could not be said to have the same bleeding risk. In addition, other previous studies on whether or not APT should be continued have often used preoperative APT‐free patients as a control group for the APT continuation group.^20^, ^21^ This was thought to be due to the limited number of patients who received APT. However, the preoperative administration of APT was considered to be associated with a high‐risk postoperative CVE, and the risk postoperative bleeding and CVE in the above setting was potentially different. Thus, we only included patients who had received APT preoperatively in this study. Few studies have compared the presence or absence of APT in a population limited to patients who were using APT preoperatively prior to laparoscopic colorectal resection. In this study, all patients who had received APT for primary prevention were included in the d‐APT group. Indeed, patients used for primary prevention may potentially have fewer serious comorbidities and serious past medical history. However, we considered that patients who had received primary prevention have been prescribed for the presence of some comorbidities with cardiovascular and/or cerebrovascular event (CVE) risk, and it was considered that patients with preoperative APT had a higher CVE risk than patients who were not prescribed APT. For that reason, we considered that patients with extremely low CVE risk were not included in this study.

The results of our study did not show a significant increase in intraoperative blood loss or postoperative hemorrhagic complications associated with continued APT. On the contrary, the occurrence of cerebral infarction was observed after the discontinuation of APT. The operation times of the two cases with postoperative non‐fatal cerebral infarction were 150 minutes respectively. In addition, postoperative pulmonary thromboembolism was only observed in one case of the d‐APT group. The operation time for the case of postoperative pulmonary infarction was 180 minutes. These operation times were not a particularly long time in this study.

Stroke is a profoundly serious and potentially fatal complication that can leave sequelae. The overall incidence of stroke in this study was 0.93% (2/214). This complication was rare; however, it could not be ignored. Of the two cases of postoperative non‐fatal cerebral infarction, one occurred in a patient who had received APT for TIA preoperatively. Thus, it was considered that this case had a high risk of developing cerebral infarction. However, the population of patients who received APT preoperatively was considered to include some patients with a high risk of developing cardiovascular and/or cerebrovascular disease. If the hemorrhagic risk was tolerable in the perioperative period, it was considered to be desirable to continue APT from the viewpoint of the risk of CVE. Laparoscopic surgery for CRC is one of the most standardized surgical procedures, and the risk of bleeding is considered to be well‐controlled. From the results of this study, it was considered desirable to continue perioperative APT in patients undergoing laparoscopic surgery for CRC.

At the discretion of the anesthesiologist, it was determined whether epidural anesthesia was performed. In this study, epidural anesthesia was not performed in c‐APT group. Epidural anesthesia was performed in cases where the anesthesiologist considered it necessary in d‐APT group. This study suggested the possibility that the continuation or discontinuation of APT may have influenced the decision whether to perform epidural anesthesia. However, this may be due to historical background. Currently, aspirin is unrestricted for time intervals before and after neuraxial puncture or catheter manipulation or removal at the recommendation of the European Anesthesiology Society.^22^ American Society of Regional Anesthesia and Pain Medicine also treats aspirin separately from other antiplatelet drugs.^23^ If these were accepted more widely, the influence of continuing aspirin on the presence or absence of epidural anesthesia may disappear.

The present study was associated with some limitations. First, this study was retrospective in nature and had a limited sample size. Second, non‐clinical hemorrhagic complications and CVE might have gone undetected because contrast‐enhanced CT was not routinely performed after surgery. Another limitation was the relatively small number of postoperative events. Therefore, further randomized prospective trials with a larger study population are needed to validate the efficacy of the continuation of APT. However, the authors believe that the findings of this study will provide a firm foundation for future studies.

In conclusion, we revealed that there was no significant difference in the surgical outcomes or postoperative complications between patients who underwent laparoscopic surgery for CRC with the continuation of APT in the perioperative period and those in whom APT was discontinued in the perioperative period. However, two patients in the d‐APT group developed non‐fatal stroke after surgery, while no patients who continued to receive APT experienced this complication. Non‐fatal stroke is a significant non‐negligible complication. Thus, it may be better to continue APT in laparoscopic surgery for CRC.

## DISCLOSURE

Conflict of Interest: The authors declare no conflict of interest in association with the present study.

Meeting Presentation: None.
